# Patient’s perception of a nurse-led Trastuzumab pathway

**DOI:** 10.3332/ecancer.2014.419

**Published:** 2014-04-10

**Authors:** Helen Roe

**Affiliations:** North Cumbria University Hospitals NHS Trust, Carlisle, CA2 7HY, UK

**Keywords:** patient pathway, nurse-led, breast cancer, Trastuzumab

## Abstract

This paper presents the development of a pathway at a cancer unit in Northern England. The pathway aimed to ensure that patients with early breast cancer who were receiving Trastuzumab did so in a safe manner. This was achieved by developing a multiprofessional pathway, which crossed traditional boundaries to ensure the patient received the best care available. This paper describes the patient’s perception of the pathway obtained through a patient satisfaction survey and suggestions for its future direction.

## Background

The National Institute for Clinical Excellence issued its guidance on the use of Trastuzumab in early breast cancer and stressed the importance of cardiac monitoring for this patient group, which would involve either three monthly echocardiograms (echo) or radionuclide (MUGA) imaging [1]. This monitoring is essential due to the fact that Trastuzumab has the potential to decrease the left ventricular ejection fraction and cause possible heart failure [2]. The organisation where the survey took place uses echoes as a standard form of monitoring and only uses MUGA’s where images are unacceptable on echocardiogram, i.e., due to an implant.

**Figure 1:**
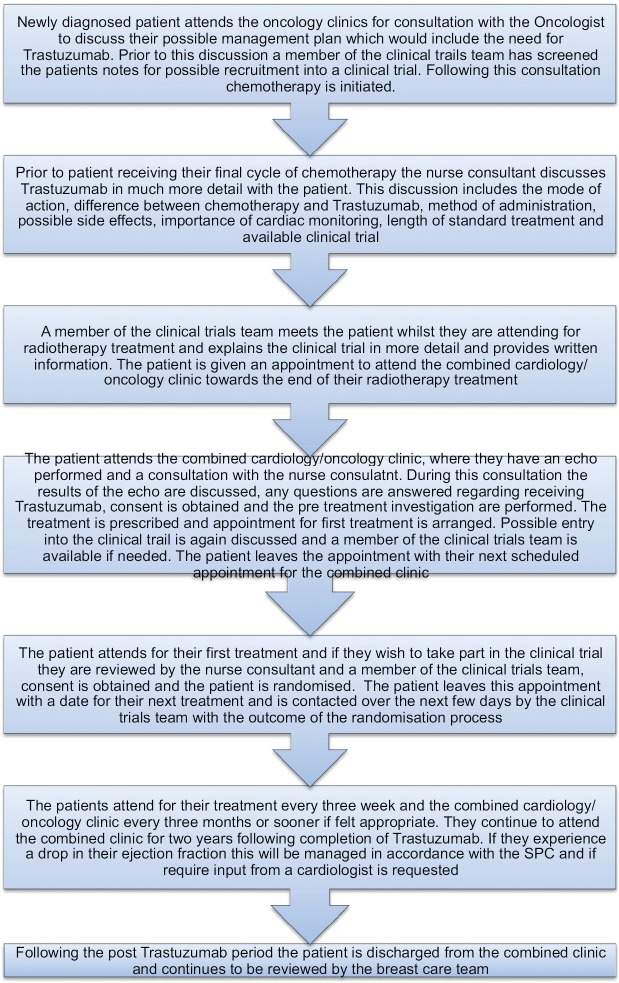
Patient pathway.

The nurse consultant for breast care led the development of a multiprofessional pathway back in 2010. The patient is commenced on the pathway (Figure 1) when the potential benefits of receiving Trastuzumab are discussed at their initial consultation with the oncologist. Further discussions take place during the other adjuvant treatments (chemotherapy and radiotherapy) by various members of the multiprofessional team. A key point in the pathway is when the nurse consultant meets the patient following their final cycle of chemotherapy. This consultation involves further explanation in more detail about Trastuzumab, including mode of action, delivery, length of treatment, possible side effects, and, importantly, the need for continued cardiac monitoring. Clinical trials are also discussed, and a member of the trials team will meet the patient during radiotherapy [3].

The pathway is followed as described in Figure 1.

The development of this pathway provided all professional groups with the opportunity to demonstrate how working together enhanced patient care [4]. One specific example of this concerns the cardiac monitoring, where a joint consultation is standard practice for patients. This pathway was reliant on professionals working across standard boundaries and sharing responsibilities for patient care and the success of the pathway.

A key factor in the rationale behind developing this pathway was the local geography and the need for patients to constantly travel for part of their treatment. In addition, there was a need to deliver as much care safely nearer the patient’s home. The main difficulty was that the organisation includes two hospitals 40 mi apart, and, initially, when the pathway was developed, echoes were only performed on one hospital site as part of a combined clinic provided by the nurse consultant and a cardiologist.

Over time, it became apparent that the number of patients accessing the pathway had remained at around a 50:50 split between the two hospitals, which meant half the patients needed to undergo an 80 mi round trip for their echo, which, in fact, could be performed locally. Increasingly, the alternative pathway was being challenged as there was an existing review clinic at the other hospital. By making this change it would save patients travelling unnecessarily and free up urgently needed capacity at the original hospital where they had recently opened a ‘heart centre,’ which was generating an increase in the cardiology workload.

Patients required admission to the inpatient ward for their initial treatment due to the required observation period after the infusion and a lack of capacity in the outpatient settings. All consequent treatments were delivered in our outpatient settings in both hospitals. When this service was initially developed a business case had been prepared and approved by the commissioners, which included provisions for reconstitution, administration, and monitoring of the patients receiving this treatment.

The cardiologist involved initially in the development and delivery of the combined cardiology/oncology clinic left, putting the service at risk, but the combined clinic has continued. The structure changed with the echoes now being performed by a cardio-physiologist, with a patient review by the nurse consultant with a diagnostic imaging cardiologist available to advise if required. The clinic has continued to receive positive verbal feedback and the changes have not compromised patient care in any way.

The demand on the service overall has increased, partly due to gastric patients also requiring cardiac monitoring whilst receiving Trastuzumab [2], along with other chemotherapy developments which are also affecting the requirements on both the pharmacy and nursing elements of the service. There were a number of options available to alter the current service but before making changes the service felt it was important to understand the patient view.

## Methodology

A patient satisfaction survey of the complete pathway was developed to determine if the pathway met the needs of the patients accessing it, and whether there were any lessons to be learnt regarding possible future developments.

The survey consisted of nine closed questions and aimed to produce quantitative data relating to the provision of information, preference for echo, clinical trial availability timing of infusions, and overall satisfaction. A free text option was also available.

With this particular survey the patients selected needed to be very specific to allow the questions to be answered appropriately, and therefore a purposeful sampling technique was used. This allowed a pre selection of patients who had insight into and knowledge of the pathway.

The survey was sent to 40 patients who were receiving Trastuzumab in the adjuvant setting, in the two-year follow-up period with a stamped addressed envelope. Of the 40 surveys sent out, 32 were returned, and the following represents the result received.

## Results

### Provision of information

**Figure 2:**
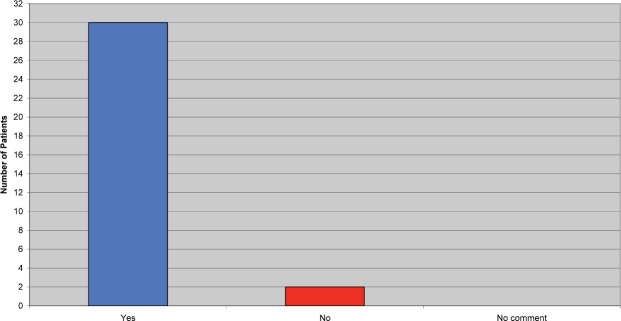
Did you receive adequate information prior to commencing Trastuzumab?

Patients were asked if they felt they received adequate information before commencing Trastuzumab. Although the majority of patients felt they did, a few patients commented they would have liked further information on side effects, how they might build up over the treatment period, and how to manage them when they occur (Figure 2).

### Where would you prefer your echocardiogram performed?

**Figure 3:**
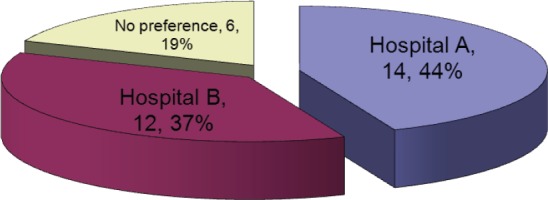
Where would you prefer your echo to be performed?

This question focused on echocardiograms, as MUGA’s were already available in both hospitals. The result to this question represented a roughly equal split between the two hospitals, which was consistent with the patient split (Figure 3).

### Where you offered the opportunity to take part in a clinical trial?

**Figure 4:**
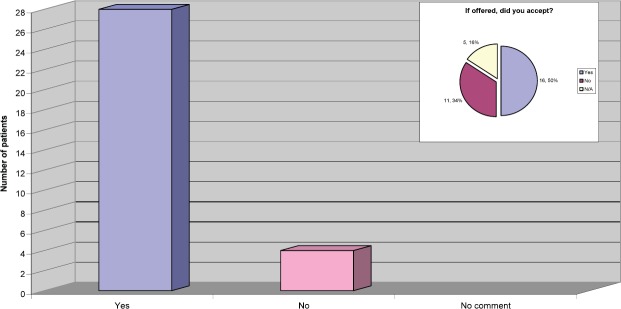
Were you offered the opportunity to take part in a clinical trial?

The response to this question was very reassuring to the team, as they had been actively screening patients for eligibility to take part in the Persephone Study [3]. The results also demonstrated that, of the patients provided with information regarding the study, half of them agreed to enter the study (Figure 4).

### Choice of place to receive Trastuzumab

**Figure 5:**
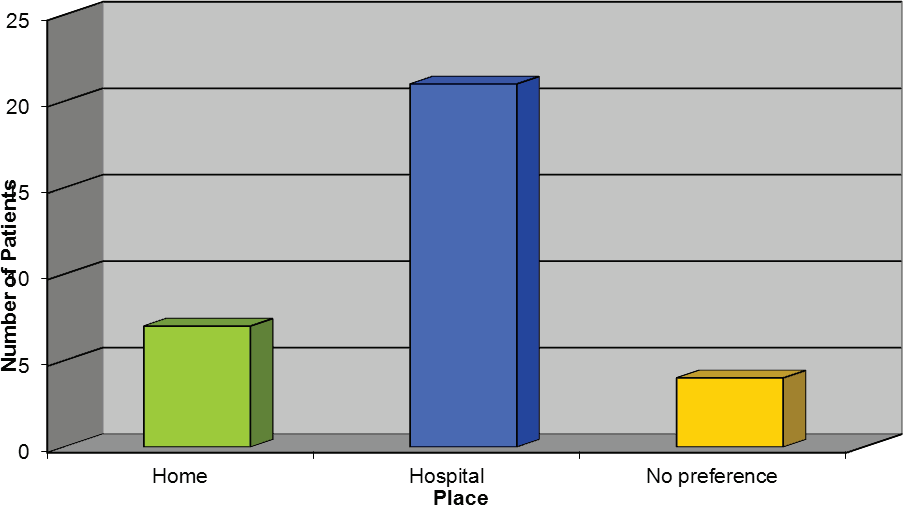
Choice of place to receive Trastuzumab.

Figure 5 demonstrates the response we received regarding where patients would like to receive their treatment, and, surprisingly, the majority of patients would rather receive it in the hospital setting. However, the author felt this should not exclude the options being discussed with patients as evidently some patients would prefer to receive their treatment this way.

### Length of the infusion time

It is nationally recognised that it is safe to alter the infusion time from 90 to 30 min if the patient tolerates the treatment and has no reactions or drop in their ejection fraction. Our practice is to deliver the first four cycles at 90 min, and then consider reducing the time to 30 min following discussion with the patient.

**Figure 6:**
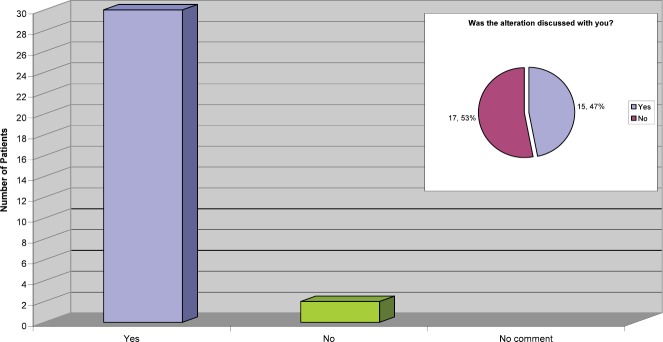
Length of infusion reduced to 30 min.

From the replies received to this question (Figure 6) it was apparent that for the majority of patients the infusion time were altered, which has the potential to release more nursing and chair time in the outpatient area. However, it was a concern that half the patients had no recollection of discussions regarding the possibility of this being an option or actually happening.

### Overall satisfaction with the pathway

**Figure 7:**
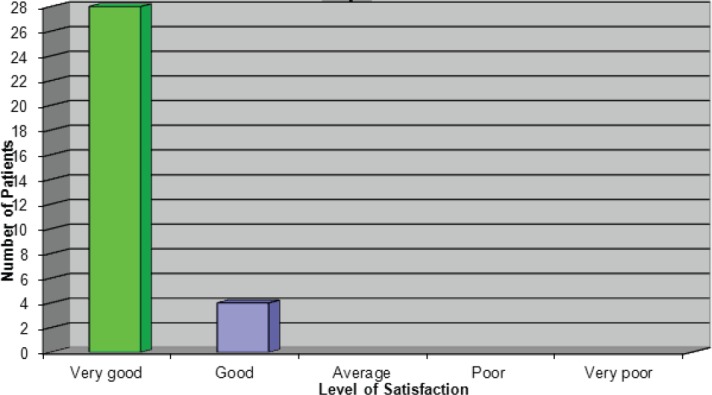
Overall how satisfied where you with the service you received?

The results to this question were very pleasing as they reiterated what the patients were expressing verbally (Figure 7). One patient who rated the service as ‘good’ stated that, had she not experienced a delay with her first treatment, she would have given the service a very good rating.

### Discussion

The good response rate to the survey provides the service with a great deal of information, which will assist in ensuring the service remains patient focused. The section for patients to make any comments they felt would help the team improve highlighted a number of themes which include:

- less travel if the combined clinic was split between the two hospitals;
- glad to see the service still running as it focuses very much on patient needs;
- attending the combined cardiology/oncology clinic is a pleasanter experience;
- staff always professional but approachable;
- glad to know my age didn’t stop me having the treatment;
- not sure if clinical trials should be offered (individual comment);
- drugs arrive late from pharmacy leading to delays for the patient, nurse and next patient who needs the chair;
- nurses need to spend time calculating the patient’s next appointment;
- service received was excellent and can’t be faulted.

## Conclusion

Following the development of the pathway and the patient feedback received, the combined cardiology/oncology clinic has now been restructured to provide a service on both hospital sites. This change has reduced the number of appointments in the original clinic. Importantly it has allowed for better utilisation of available capacity and ensured that the service has remained patient focused.

The service continues to offer and recruit patients into clinical trials and has recently been highlighted as a good recruiter into the Persephone Study [3]. Further discussions between oncology and pharmacy regarding the option of patients receiving home Trastuzumab and development of a pathway are currently taking place, especially with the new licenced indications for subcutaneous Trastuzumab [5]. The concerns around the patients not recollecting discussions regarding the infusion time being shortened have been shared with the team. The development of this nurse led pathway is one of many nurse led initiatives that have been implemented for cancer patients in the organisation, which with the support of colleagues have been a success, demonstrating that professionals working together can enhance patient care.

This nurse-led pathway has received much recognition as it incorporated the first combined cardiology and oncology clinic when it was initially implemented. In 2013, it received a national award as an example of celebrating excellence regarding a development that is focused around the patient and improving their experience.

## Possible limitations

The number of patients invited to take part in this survey may be viewed as insufficient to formulate any major conclusions. Another limitation may be the selection of patients.

It is acknowledged that larger centres offering a different treatment pathway may experience difficulties replicating the survey, however, it met the needs for evaluating the pathway in the local organisation. There has been interest in both the survey and pathway from other organisations within England who would like to adapt this work to evaluate their local services.

## Conflicts of interest

The author has no conflicts of interest to declare.
